# Prevalence and characteristics of psychiatric morbidity treated in specialized health care in a nationwide cohort of people with newly diagnosed Alzheimer's disease

**DOI:** 10.1111/acps.13423

**Published:** 2022-03-16

**Authors:** Aleksi Alastalo, Anna‐Maija Tolppanen, Miika Nietola, Marianne Haapea, Jouko Miettunen, Sirpa Hartikainen, Erika Jääskeläinen

**Affiliations:** ^1^ Center for Life Course Health Research University of Oulu Oulu Finland; ^2^ Medical Research Center Oulu Oulu University Hospital and University of Oulu Oulu Finland; ^3^ School of Pharmacy University of Eastern Finland Kuopio Finland; ^4^ Department of Psychiatry University of Turku and Turku University Hospital Turku Finland; ^5^ Department of Psychiatry Oulu University Hospital Oulu Finland

**Keywords:** Alzheimer disease, mental disorders, prodromal symptoms, psychotropic drugs

## Abstract

**Objective:**

Psychiatric disorders have been implied as both risk factors and prodromal symptoms of Alzheimer's disease (AD). A better understanding of the history of psychiatric morbidity in people with AD may aid with understanding this relationship and highlight challenges in diagnosing AD in people with concomitant psychiatric disorders.

**Methods:**

Medication use and Alzheimer's disease (MEDALZ) study is a nationwide register‐based cohort of people (*n* = 70,718) who received a clinically verified AD diagnosis in Finland in 2005–2011 and were community‐dwelling at the time of diagnosis. The study population was divided into four groups based on psychiatric morbidity treated in specialized health care. We characterized the groups using data of psychiatric and somatic illnesses, psychotropic drug use, and socioeconomic factors and investigated factors associated with prodromal AD.

**Results:**

Altogether, 4.3% of cohort members had a psychiatric diagnosis at least five years before AD diagnosis, 3.1% had a psychiatric diagnosis only up to five years before AD diagnosis, and 1.1% had a psychiatric diagnosis both less and more than 5 years before AD. Belonging to the Prodromal group (psychiatric diagnosis within 5 years before AD diagnosis) was most strongly associated with substance abuse (RR 65.06, 95%CI 55.54–76.22). Other associated factors with the Prodromal group were female gender, use of psychotropics, stroke, and asthma/COPD.

**Conclusion:**

Substance abuse and psychotropic drug use are common five years before AD diagnosis. These can be potential markers of possible prodromal symptoms of AD and should be acknowledged in clinical work.

**Significant Outcomes and limitations**

- When new onset substance abuse, psychiatric disorders or the need for psychotropic medication appear at an older age, one should consider prodromal AD behind the symptoms.
- The limitation of this study is the restriction of psychiatric diagnoses only from specialized health care.
- Therefore, the less severe psychiatric disorders managed entirely in primary health care were not recognized in this study.

## 1 INTRODUCTION

Cognitive disorders are the most common cause of loss of independent functioning in the older population. Currently, an estimated 44 million people worldwide have a progressive cognitive disorder or dementia, of which Alzheimer's disease (AD) accounted for 50–75% of the cases. Dementia imposes increasing costs on countries, municipalities, families, and individuals. By the year 2050, it is estimated, that the number of people with cognitive disorders is going to be more than triple.^1^

Alzheimer's disease (AD) is a progressive neurodegenerative disease in which the disease process can precede the diagnosis by years or even decades.^2^, ^3^ Different symptoms, including psychiatric and neuropsychiatric symptoms, may manifest during the prodromal stage,^4^ and they may result in need of psychiatric treatment. On the other hand, recognizing and differentiating prodromal symptoms of AD from psychiatric disorders in psychiatric treatment services is essential, since AD may hide behind complex psychiatric symptomatology, and this may cause a delay in receiving further examination and appropriate treatment.

Furthermore, this complexity is illustrated by the debate on, whether psychiatric illnesses are risk factors of AD or whether they are symptoms of prodromal AD. There are some studies on the association between depression and AD,^5^, ^6^, ^7^, ^8^, ^9^ and very few on other mental disorders, such as anxiety disorders^8^ and psychoses.^10^ Depression and anxiety seem to increase the risk of AD, but it is unknown if they are causal risk factors or prodromal symptoms of AD and its neurodegenerative process.^7^, ^8^ It is also suggested that psychotic disorders increase the risk of AD and that psychosis symptoms at the prodromal stage of AD are more common than supposed based on previous evidence.^10^ However, the interpretation of the studies and the role of psychiatric symptomatology as risk factors or prodromal symptoms of AD is complicated by methodological issues such as cross‐sectional study design and narrow timeline between psychiatric symptoms and AD onset.^11^, ^12^, ^13^ In addition, earlier studies have been more focused on specific disorders, while the wider understanding of the incidence of these diagnoses has been studied less. Further studies could aid in earlier recognition of AD and this way enable earlier treatment of AD.^14^

### 1.1 Aims of the study

This study aimed to characterize the history of psychiatric disorders that required specialized health care in a nationwide cohort of people with a clinically verified AD diagnosis. We also studied the derived groups based on whether psychiatric morbidity was diagnosed during the prodromal phase or earlier and did these groups differ from each other based on their clinical characteristics.

## 2 MATERIALS AND METHODS

### 2.1 Medication use and Alzheimer's disease (MEDALZ) cohort

Medication use and Alzheimer's disease (MEDALZ) study is a nationwide register‐based cohort of 70,718 people who received a clinically verified diagnosis of AD in Finland in 2005–2011 and were community‐dwelling at the time of diagnosis. AD diagnoses were identified from the Special reimbursement register maintained by the Finnish Social Insurance Institution (SII). AD diagnosis in Finland must be verified by appropriate medical examination including verification according to AD’s DSM‐IV and NINCS‐ADRDA criteria, CT/MRI scan, the differential diagnosis between other dementing diseases, and confirmation of AD diagnosis by a neurologist or geriatrician. Additional data has been retrieved from the following national registers: SII prescription register (medication use; 1995–2012) and special reimbursement register (chronic comorbidities; 1972 −2011), the National Institute for Health and Welfare Care Register for Health Care (diagnoses of psychiatric disorders and somatic illnesses, hospitalizations 1972–2011) and Statistics Finland mortality register (2005–2011) and socioeconomic data (1970–2011). A more detailed description is available in the cohort profile.^15^

### 2.2 Psychiatric morbidity

Data on psychiatric morbidity was obtained from the Care Register for Health Care (inpatient stays 1972–2011, policlinic visits on specialized health care 1998–2011). Thus, this data does not include diagnoses from primary health care but only more severe cases, which required specialized health care. Persons were categorized into four groups according to whether they had admissions or visits in specialized health care with the diagnosis code of psychiatric disorders (excluding dementia) before AD diagnosis. The prevalence of psychiatric diagnoses in different groups was also studied in diagnostic subcategories. The included ICD‐10 codes (1996 onwards) were F20‐F69 and F90‐F99. The included ICD‐9 codes (1987–1995) were 295–298, 300–301, 2071–3075, 312 and 313–314. The included ICD‐8 codes (1972–1986) were 295–301, 303–308, and 7902. The search was restricted to main diagnosis codes.

The four groups were defined as follows: No diagnosis (no psychiatric diagnosis in specialized health care, *n* = 64,703), Previous diagnosis (psychiatric diagnosis more than 5 years before AD, *n* = 3009), Prodromal (psychiatric diagnosis up to five years before AD, *n* = 2222), Chronic (psychiatric diagnosis both less and more than 5 years before AD, *n* = 784). The choice of a five‐year time window is based on clinical experience and previous studies.^4^, ^10^, ^16^ According to six longitudinal cohort studies combined (*n* = 3268) the mean duration of prodromal Alzheimer's disease ranged from 3.5 to 4.6 years.^16^

### 2.3 Sociodemographic and clinical characteristics

The following sociodemographic and clinical characteristics were analyzed (Table S1): sex, occupational social class, asthma / chronic obstructive pulmonary disease (COPD), cardiovascular disease, diabetes, epilepsy, stroke, substance abuse, psychotropic drug use (antidepressant, antipsychotics and benzodiazepines and related drugs) and opioid use. Psychotropic drug use and opioid use were defined based on ATC codes. All information above was collected from the registers mentioned earlier. Detailed definition of sociodemographic and clinical characteristic covariates is available in Table S1.

### 2.4 Statistical analyses

Characteristics of four groups were described by appropriate statistical methods (frequencies with percentages; means with 95% confidence interval (CI); medians with interquartile range (IQR)). Differences in categorical variables were assessed with the Chi‐square test. Differences in the distribution of normally distributed continuous variables between the groups were compared with linear regression. Differences in hospital days (skewed distribution) were compared with the Kruskal–Wallis test and Mann–Whitney test. Associations between characteristics and belonging to the prodromal group were presented as relative risks (RR) with CI and assessed using logistic regression. All statistical analyses were conducted with Stata MP14.0.

## 3 RESULTS

Altogether 6015 (8.5%) of people diagnosed with AD had a psychiatric diagnosis before the diagnosis of AD. Nearly half of these belonged to the Previous diagnosis ‐group (psychiatric diagnosis at least five years before AD diagnosis, *n* = 3009, 4.3%), 2222 (3.1%) to the Prodromal group (psychiatric diagnosis only up to 5 years before AD diagnosis) and 784 (1.1%) persons belonged to the Chronic ‐group (psychiatric diagnosis both less and more than 5 years before AD) (Table 1).

**TABLE 1:** Sociodemographic characteristics, somatic diseases, and substance abuse in groups based on psychiatric morbidity

Variable	No diagnosis (*n* = 64,703)	Previous diagnosis (*n* = 3009)	Chronic (*n* = 784)	Prodromal (*n* = 2222)	*p*‐value
Sex, *n* (%)					
Women	42,069 (65.0)	1979 (65.8)	531 (67.7)	1537 (69.2)	<0.001
Men	22,634 (35.0)	1030 (34.2)	253 (32.3)	685 (30.8)	
Age at AD diagnosis, mean (95% Cl)	80.2 (80.2–80.3)	78.8 (78.5–79.0)	75.9 (75.3–76.4)	78.6 (78.3–78.9)	<0.001
Socioeconomic status, *n* (%)					
Managerial/professional	13,614 (21.0)	509 (16.9)	132 (16.8)	437 (19.7)	<0.001
Office	5472 (8.5)	211 (7.0)	77 (9.8)	213 (9.6)	
Farming/forestry	12,360 (19.1)	570 (18.9)	139 (17.7)	370 (16.7)	
Sales / industrial / cleaning	27,468 (42.5)	1374 (45.7)	327 (41.7)	979 (44.1)	
Unknown	5789 (8.9)	345 (11.5)	109 (13.9)	223 (10.0)	
Asthma or COPD, *n* (%)	6717 (10.4)	410 (13.6)	88 (11.2)	266 (12.0)	<0.001
Cardiovascular disease, *n* (%)	32,155 (49.7)	1565 (52.0)	334 (42.6)	1117 (50.3)	<0.001
Diabetes, *n* (%)	11,017 (17.0)	630 (20.9)	162 (20.7)	400 (18.0)	<0.001
Epilepsy, *n* (%)	958 (1.5)	115 (3.8)	34 (4.3)	36 (1.6)	<0.001
Stroke, *n* (%)	6097 (9.4)	367 (12.2)	76 (9.7)	248 (11.2)	<0.001
Substance abuse, *n* (%)					
Before prodromal only	2580 (4.0)	768 (25.5)	73 (9.3)	81 (3.6)	<0.001
Both	21 (0.0)	30 (1.0)	152 (19.4)	59 (2.7)	
Prodromal only	238 (0.4)	8 (0.3)	27 (3.4)	449 (20.2)	
Hospital day accumulation due to the somatic diagnosis, median (IQR)					
>5 years before AD diagnosis	7 (1–20)	14 (3–37)	11 (1–34)	7 (1–21)	0.001
<5 years before AD diagnosis	9 (1–30)	15 (2–44)	22 (4–66)	22 (4–58)	0.001

Abbreviations: COPD, Chronic obstructive pulmonary disease; IQR, Interquartile range; CI, Confidence interval; AD, Alzheimer's disease; Prodromal, A period of five years before the diagnosis of Alzheimer's disease; Both, During and before the prodromal period.

The age at AD diagnosis was the highest in the No diagnosis ‐group (80.2 years, 95%CI 80.2–80.3) and the lowest in the Chronic ‐group (75.9 years, 95%CI 75.3–76.4). The proportion of women was the highest in the Prodromal group (69.2%). There were no clinically significant differences in the incidence of somatic diseases between the groups in the five‐year period before AD diagnosis, however, during this period, the No diagnosis ‐group had fewer hospital days due to somatic diagnosis compared to other groups (Table 1).

The Chronic ‐group had the highest proportion of psychiatric disorders: altogether 51.9% of them had non‐psychotic depression and 38.4% other psychoses than schizophrenia before AD diagnosis. Non‐psychotic depression was also the most common psychiatric disorder (40.8%) in the previous diagnosis ‐group. The most common psychiatric diagnoses in the Prodromal group were non‐psychotic depression (37%), schizophrenia, delusional disorder, or other related psychoses (21%), other psychosis (19%), and anxiety disorder (12%) (Table 2). Majority of persons with schizophrenia, delusional disorder, or other related psychosis in the Prodromal group, had delusional disorder (ICD‐10: F22, *n* = 417). Only 34 patients had schizophrenia (ICD‐10: F20), three had shared psychotic disorder (ICD‐10: F24) and six had schizoaffective disorder (ICD‐10: F25).

**TABLE 2:** Psychiatric main diagnoses and psychiatric hospitalization days in the groups

Variable	Previous diagnosis (*n* = 3009)	Chronic (*n* = 784)	Prodromal (*n* = 2222)	*p*‐value
Anxiety, *n* (%)				
Before prodromal only	505 (16.8)	82 (10.5)	—	<0.001
Both	—	48 (6.1)	—	
Prodromal only	—	54 (6.9)	258 (11.6)	
Schizophrenia, delusional disorder, or other related psychosis, *n* (%)				
Before prodromal only	421 (14.0)	39 (5.0)	—	<0.001
Both	—	175 (22.3)	—	
Prodromal only	—	60 (7.7)	460 (20.7)	
Other psychosis, *n* (%)				
Before prodromal only	552 (18.3)	154 (19.6)	—	<0.001
Both	—	87 (11.1)	—	
Prodromal only	—	60 (7.7)	419 (18.9)	
Non‐psychotic bipolar disorder, *n* (%)				
Before prodromal only	102 (3.4)	33 (4.2)	—	<0.001
Both	—	34 (4.3)	—	
Prodromal only	—	32 (4.1)	53 (2.4)	
Non‐psychotic depression, *n* (%)				
Before prodromal only	1229 (40.8)	128 (16.3)	—	<0.001
Both	—	190 (24.2)	—	
Prodromal only	—	89 (11.4)	811 (36.5)	
Other mood disorder, *n* (%)				
Before prodromal only	13 (0.4)	5 (0.6)	—	<0.001
Both	—	2 (0.3)	—	
Prodromal only	—	5 (0.6)	19 (0.9)	
Any other, *n* (%)				
Before prodromal only	1029 (34.2)	63 (8.0)	—	<0.001
Both	—	113 (14.4)	—	
Prodromal only	—	68 (8.7)	677 (30.5)	
Hospital day accumulation due to the psychiatric diagnosis, median (IQR)				
>5 years before AD diagnosis	0 (0–11)	15 (0–66)	—	<0.001
<5 years before AD diagnosis	—	26 (8–69)	14 (4–40)	<0.001

Abbreviations: AD, Alzheimer's disease; Both, During and before prodromal period; CI, Confidence interval; IQR, Interquartile range; Prodromal, A period of five years before the diagnosis of Alzheimer's disease.

During the prodromal period use of all psychotropics was most common in the Prodromal group. Similarly, the prevalence of psychotropic use before the prodromal period was the most common in the previous diagnosis ‐group and the use of psychotropics was most common in the Chronic ‐group when looking at the time before and during the prodromal period (Table 3).

**TABLE 3:** Use of psychotropic medication and opioids only before prodromal time, only during the prodromal time, or during both periods since 1995 in different diagnosis groups. Prodromal =a period of five years before the diagnosis of AD

Variable	No diagnosis (*n* = 64,703)	Previous diagnosis (*n* = 3009)	Chronic (*n* = 784)	Prodromal (*n* = 2222)	*p*‐value
Antidepressant, *n* (%)					
Before prodromal only	3648 (5.6)	356 (11.8)	61 (7.8)	91 (4.1)	<0.001
Both	7105 (11.0)	1192 (39.6)	445 (56.8)	621 (27.9)	
Prodromal only	10,309 (15.9)	339 (11.3)	111 (14.2)	801 (36.0)	
Antipsychotics, *n* (%)					
Before prodromal only	970 (1.5)	322 (10.7)	57 (7.3)	51 (2.3)	<0.001
Both	937 (1.4)	683 (22.7)	425 (54.2)	145 (6.5)	
Prodromal only	5279 (8.2)	279 (9.3)	159 (20.3)	982 (44.2)	
Benzodiazepines and related drugs, *n* (%)					
Before prodromal only	5315 (8.2)	386 (12.8)	64 (8.2)	130 (5.9)	<0.001
Both	16,137 (24.9)	1488 (49.5)	505 (64.4)	983 (44.2)	
Prodromal only	6353 (9.8)	231 (7.7)	100 (12.8)	459 (20.7)	
Opiates, *n* (%)					
Before prodromal only	5921 (9.2)	344 (11.4)	75 (9.6)	220 (9.9)	<0.001
Both	3514 (5.4)	277 (9.2)	73 (9.3)	184 (8.3)	
Prodromal only	6572 (10.2)	361 (12.0)	76 (9.7)	241 (10.8)	

Abbreviations: AD, Alzheimer's disease; Both, During and before the prodromal period; Prodromal, A period of five years before the diagnosis of Alzheimer's disease.

### 3.1 Factors associated with belonging to the Prodromal group

Associations between characteristics and likelihood of belonging to the Prodromal group are presented in Table 4. The incidence of substance abuse during the prodromal period was strongly associated with belonging to the Prodromal group (RR 65.1, 95%CI 55.5–76.2) when compared to the other three groups. In stratified analyses, this association was stronger in men (RR 100.4, 95%CI 80.4–125.3) than in women (RR 55.5, 95%CI 43.6–70.6). The use of antipsychotics, antidepressants, and benzodiazepines or benzodiazepine‐related drugs were significantly associated with the Prodromal group when compared to non‐users of these drugs in all time windows but especially during the prodromal period. Stroke and asthma/COPD were also significantly associated with the Prodromal group, even though confidence intervals were near one.

**TABLE 4:** Association between characteristics and belonging to the Prodromal group

Variable	Prodromal group (*n* = 2222)	Other groups (*n* = 68,496)	Crude RR (95% CI)
Sex, *n* (%)			
Women	1537 (69.2)	44,579 (65.1)	1.00 (reference)
Men	685 (30.8)	23,917 (34.9)	0.83 (0.76–0.91)
Socioeconomic status, *n* (%)			
Managerial/professional	437 (19.7)	14,255 (20.8)	1.00 (reference)
Office	213 (9.6)	5760 (8.4)	1.21 (1.02–1.42)
Farming / forestry	370 (16.7)	13,069 (19.1)	0.92 (0.80–1.06)
Sales / industrial / cleaning	979 (44.1)	29,169 (42.6)	1.09 (0.98–1.23)
Unknown	223 (10.0)	6243 (9.1)	1.17 (0.99–1.37)
Asthma or COPD, *n* (%)	266 (12.0)	7215 (10.5)	1.16 (1.01–1.32)
Cardiovascular disease, *n* (%)	1117 (50.3)	34,054 (49.7)	1.02 (0.94–1.11)
Diabetes, *n* (%)	400 (18.0)	11,809 (17.2)	1.05 (0.94–1.18)
Epilepsy, *n* (%)	36 (1.6)	1107 (1.6)	1.00 (0.72–1.40)
Stroke, *n* (%)	248 (11.2)	6540 (9.5)	1.19 (1.04–1.36)
Substance abuse, *n* (%)			
None	1633 (73.5)	64,599 (94.3)	1.00 (reference)
Before prodromal only	81 (3.6)	3421 (5.0)	0.94 (0.75–1.17)
Both	59 (2.7)	203 (0.3)	11.50 (8.57–15.43)
Prodromal only	449 (20.2)	273 (0.4)	65.06 (55.54–76.22)
Antidepressant use, *n* (%)			
No use	709 (31.9)	44,930 (65.6)	1.00 (reference)
Before prodromal only	91 (4.1)	4065 (5.9)	1.42 (1.14–1.77)
Both	621 (27.9)	8742 (12.8)	4.50 (4.03–5.03)
Prodromal only	801 (36.0)	10,759 (15.7)	4.72 (4.26–5.23)
Antipsychotic use, *n* (%)			
No use	1044 (47.0)	59,385 (86.7)	1.00 (reference)
Before prodromal only	51 (2.3)	1349 (2.0)	2.15 (1.62–2.86)
Both	145 (6.5)	2045 (3.0)	4.03 (3.37–4.82)
Prodromal only	982 (44.2)	5717 (8.3)	9.77 (8.92–10.70)
Benzodiazepine and related drug use, *n* (%)			
No use	650 (29.3)	37,917 (55.4)	1.00 (reference)
Before prodromal only	130 (5.9)	5765 (8.4)	1.32 (1.09–1.59)
Both	983 (44.2)	18,130 (26.5)	3.16 (2.86–3.50)
Prodromal only	459 (20.7)	6684 (9.8)	4.01 (3.54–4.53)
Opiate use, *n* (%)			
No use	1577 (71.0)	51,283 (74.9)	1.00 (reference)
Before prodromal only	220 (9.9)	6340 (9.3)	1.13 (0.98–1.30)
Both	184 (8.3)	3864 (5.6)	1.55 (1.32–1.81)
Prodromal only	241 (10.8)	7009 (10.2)	1.12 (0.97–1.28)

Abbreviations: Both, During and before prodromal period; COPD, Chronic obstructive pulmonary disease; Prodromal, A period of five years before the diagnosis of Alzheimer's disease; RR, Risk ratio.

## 4 DISCUSSION

Our nationwide register‐based study of people with clinically verified AD diagnoses identified four different subgroups based on the history of psychiatric diagnoses. People who received their first diagnosis of depression, schizophrenia, delusional disorder, other related psychoses or other psychotic disorder, or anxiety disorder in the five‐year time window before AD diagnosis, possibly manifest prodromal symptoms rather than actual psychiatric disorders. Worryingly, substance abuse and the use of psychotropic drugs were more common in this group.

### 4.1 Psychiatric morbidity in the prodromal phase of AD

In our study, 36.5% received a new diagnosis of depression during the prodromal time. Our findings of the Prodromal group are in line with previous studies that have found depression to be part of the prodromal phase of AD.^6^, ^7^, ^17^ In addition, a 28‐year follow‐up study did not find an association between depressive symptoms and the risk of dementia.^9^ Chan et al.^5^ showed that late‐onset (age over 65 years) major depressive disorder is a part of the prodromal phase of AD. It should be noted that also Tapiainen et al.^17^ studied the association between psychiatric morbidity and risk of AD in a study population with a minor overlap with the present study, as some people with AD diagnosis from 2005 were also included in that study. However, the overlap is minor, and the important difference between that and the current study is in the design: Tapiainen et al.^17^ examined psychiatric morbidity and psychiatric disorders as AD risk factors in a case‐control setting, while we examined the prevalence of psychiatric morbidity in people with AD and assessed characteristics associated with newly diagnosed psychiatric disorders during the prodromal phase.

Numerous mechanisms may explain the manifestation of psychiatric disorders in the prodromal phase of AD, such as inflammation, hypothalamic‐pituitary‐adrenal axis function, and stress‐induced changes in the body. It may be, that psychiatric symptoms and disorders are related to the neurodegenerative process behind the development of AD or that they are an independent phenomenon.^8^

It has been reported that depression and anxiety in patients with mild cognitive impairment independently predict AD onset.^18^ As many as 97% of patients with diagnosed AD are suggested to have neuropsychiatric symptoms, most frequently apathy and depression.^19^ Gallagher et al.^18^ presented a follow‐up study of 167 patients with mild cognitive impairment, in which 76% of the patients had at least one neuropsychiatric symptom, the most common ones being anxiety, affective disturbance, and aggressiveness. However, neuropsychiatric symptoms like agitation and aggression that are present already in the prodromal phase of AD might have been considered as psychotic symptoms and lead to a psychiatric diagnosis, which was common in the Prodromal group in our study. This is supported by previous findings^20^, ^21^ stating that initiation of psychotropic drug use is frequent already in the prodromal phase of AD. Thus, there may be a higher risk for unnecessary or even inappropriate pharmacotherapy and disadvantages of polypharmacy.

New‐onset schizophrenia at an older age is very rare; however, somewhat similar figures than ours have been found in previous studies with an older study population.^17^, ^22^ In our study still 20.7% of the Prodromal group had a new diagnosis of schizophrenia, delusional disorder, or other related psychoses and 18.9% had a new diagnosis of other psychosis during 5 years before the diagnosis of AD. The incidence of schizophrenia, delusional disorder, or other related psychoses in the prodromal group is high and that is an interesting finding. However, it should be noted that in our study most of the diagnoses were delusional disorders and only a small number of persons had incident schizophrenia diagnosis in the prodromal time window. One possible explanation for a high proportion of persons with these newly diagnosed psychiatric disorders is that the ongoing AD disease process may trigger these disorders. Neuropsychiatric symptoms of prodromal AD may also be misdiagnosed as mental disorders due to the complex symptomatology of prodromal AD. Differential diagnostics of AD and other cognitive disorders, such as Lewy body dementia and frontotemporal dementia, are highly complex.^1^, ^17^, ^23^ In addition, our study criteria for AD diagnosis required that symptoms and clinical findings were mainly due to Alzheimer's disease, but persons can have mixed etiology with symptoms of Lewy body dementia.

### 4.2 Substance abuse in the prodromal phase of AD

Substance abuse (mostly alcohol abuse in this study) was initiated in the prodromal period by every fifth patient in the Prodromal group and it was more common among men than women. The high proportion of persons with substance abuse might be explained by the neurodegenerative process of AD,^1^ which may lead to problems in self‐control, impulsivity, decision‐making already and neuropsychiatric symptoms already before the diagnosis. Hypothetically this may even lead to substance abuse. Alcohol might be also used as self‐medication for complex symptoms. It also seems that in the Prodromal group, individuals who had substance abuse before the prodromal period tend to continue it also in the prodromal period. In line with this, De Oliveira et al.^24^ pointed out that people with AD who used alcohol earlier were the most likely to continue it in later life.

### 4.3 Other characteristics associated with belonging to the Prodromal group

Earlier diagnosis age in the prodromal group may reflect the fact that people with a recent mental illness are currently in treatment, which leads to an earlier diagnosis of AD. This may reflect Berkson's bias. The common use of psychotropic drugs in our sample is most probably an indicator of psychiatric symptoms needing treatment, which is in line with the finding of the high occurrence of psychiatric diagnosis during the prodromal period. However, the long‐term use of benzodiazepines is associated also with an increased risk of dementia.^25^

In our study, the socioeconomic position was quite similar in all groups except in the No diagnosis ‐group, in which a larger proportion was from a higher occupational social class. This could speak in favor of higher educated people having fewer psychiatric comorbidities.^26^

In our study, stroke was associated with belonging to the Prodromal group. Stroke has previously been associated with AD in general. It may be due to a common inflammatory and vascular factor underlying stroke, AD, and for example depression.^27^ Stroke can also cause or be a trigger for depression.^28^ Asthma/COPD was slightly associated with belonging to the Prodromal group. Previous studies have shown that hypoxemia correlates with neuropsychological impairment.^29^

### 4.4 Clinical implications

Prodromal AD is complex and identifying it may be difficult. It is hard to determine which psychiatric symptoms occur alone and which are related to prodromal AD. Our findings that prodromal AD is associated with substance abuse and psychotropic drug use may help identify these individuals. When new onset substance abuse, psychiatric disorders and the need for psychotropic medication appear at an older age, one should consider prodromal AD behind the symptoms.

People with AD are a heterogenic group of individuals with complex symptomatology. The relationship between AD and psychiatric morbidities is complex and the effect of these comorbidities in diagnosing AD as well as prognosis after AD diagnosis remains unclear. Psychiatric symptoms can complicate the identification and diagnosis of AD and misdiagnosis may delay receiving appropriate treatment. Better recognition of these persons could improve their treatment, avoiding unnecessary psychotropic prescription and perhaps even improve the quality of life.

### 4.5 Strengths and limitations of this study

The strength of this study is that the cohort includes almost all newly diagnosed AD cases in Finland in 2005–2011. In general, Finnish registers are known to have good validity. In this study, it was possible to study the entire spectrum of psychiatric disorders in the same data set. In addition, the time period for assessing psychiatric diagnosis was long (over 30 years). The characteristics that were associated with belonging to the prodromal group are relevant to clinical work and can be influenced.

Limitations of this study are that our sample included only psychiatric diagnoses based on hospitalizations and specialized healthcare outpatient visits, excluding less severe psychiatric disorders from primary health care. It should be noted that the prevalence of psychiatric disorders is likely underestimated because primary healthcare diagnoses are not included in the registry data. This presumably biases the assessment of the true levels of the psychopathology underlying the AD process and can have an impact on the findings. This is a well‐known weakness in registry data. As mentioned earlier, milder neuropsychiatric symptoms are very common in Alzheimer's disease and are likely to occur before the diagnosis of AD earlier and to a larger extent than psychiatric disorders requiring specialized health care. Thus, groups based on psychiatric diagnoses most probably would have been different if primary healthcare diagnoses were also included and our results are representative of specialized healthcare‐treated diagnoses only. On the other hand, the validity of specialized healthcare diagnoses is generally commendable.^30^ For example, the validity of schizophrenia diagnoses in the hospital discharge register has been previously demonstrated to be good.^31^ Medication use information in this study was available only since the year 1995, on the other hand, each participant had medication use information for at least 10 years before diagnosis. It should be noted that there may be statistically significant differences in large data, even if the differences are not clinically significant. We acknowledge that the definition of substance abuse in this study has limitations due to applied data sources. On the other hand, the substance abuse variable also included the reason for admission, which helped to better identify individuals with substance abuse. The substance abuse variable represents mostly alcohol abuse in our sample due to the old age of the study population, clinical experience has shown that other addictive substances or narcotics are less commonly used by older people.

Finally, we acknowledge that information on several interesting factors that could have been investigated as risk factors, including lifestyle factors, social network, and life circumstances were not available, but this is unlikely to have implications for our primary aim, the characterization of the prevalence of different psychiatric comorbidities in different time windows before AD diagnosis. To counterbalance this common limitation in register‐based studies, our study provides information on AD and psychiatric disorders treated in specialized health care requiring hospitalization in a general population during over 30‐year follow‐up, which would be practically impossible to evaluate in a clinical study.

## CONFLICT OF INTEREST

None.

### PEER REVIEW

The peer review history for this article is available at https://publons.com/publon/10.1111/acps.13423.

## Supporting information

Table S1

## Data Availability

Research data are not shared.
