# Supplementary material for: Prevalence and characteristics of psychiatric morbidity treated in specialized health care in a nationwide cohort of people with newly diagnosed Alzheimer's disease
Source: Acta Psychiatr Scand. 2022 Mar 16;145(5):507–16. doi: 10.1111/acps.13423 (PMC9311732; doi:10.1111/acps.13423)
Supplement: Supplementary file 1 — Table S1 [file ACPS-145-507-s001.docx]

Supplementary Table 1. Variables, data sources, and years.

| **Variable** | **Data sources & coding** | **Years** |
| --- | --- | --- |
| **Asthma/COPD** | Special reimbursement register |  |
|  | Code 203 | From 1972 until AD diagnosis |
|  | Care register for health care |  |
|  | ICD-10: J44-J46 | From 1995 until AD diagnosis |
|  | ICD-9: 4912A, 493, 4690A | 1987-1994 |
|  | ICD-8: 493 | 1972-1986 |
| **Substance abuse** | Prescription register |  |
|  | ATC codes N07BB, N07BC | 1995 until AD diagnosis |
|  | Care register for health care |  |
|  | Reason for admission: (33,71,72,73,74,75) | From 1995 until AD diagnosis |
|  | ICD-10: F1*, K860, K70, G621, G312, G721, I426, K292, R78 | From 1995 until AD diagnosis |
|  | ICD-9: 291, 292, 303, 304, 305, 3575, 3594A, 4255A, 5353A, 5770D-F, 5771C, 5710A, 5711A, 5712A, 5713X | 1987-1994 |
|  | ICD-8: 291, 303, 304, 57100, 57101, 57700-57708 | 1972-1986 |
| **Cardiovascular disease** | Special reimbursement register |  |
|  | Codes 205, 206, 213, 280, 201 | From 1972 until AD diagnosis |
| **Diabetes** | Prescription register |  |
|  | ATC code A10 excluding A10BX01(guar gum) | From 1995 until AD diagnosis |
|  | Special reimbursement register |  |
|  | Code 103 | From 1972 until AD diagnosis |
| **Epilepsy** | Special reimbursement register |  |
|  | Code 111 | From 1972 until AD diagnosis |
| **Stroke** | Care register for health care |  |
|  | ICD-10: I60-I64, I69 | From 1995 until AD diagnosis |
|  | ICD-9: 430-432, 4330A, 4331A, 4339A, 4340A, 4341A, 4349A, 4360, 4380 | 1987-1994 |
|  | ICD-8: 430-434 | 1972-1986 |
| **Anxiety** | Care register for health care |  |
|  | ICD-10: F40-F44 | From 1996 until AD diagnosis |
|  | ICD-9: 3000-3003, 3006-3009 | 1987-1995 |
|  | ICD-8: 3000-3003, 3005-3009 | 1968-1986 |
| **Schizophrenia, delusional disorder, or other related psychosis** | Care register for health care |  |
|  | ICD-10: F20, F22, F24, F25 | From 1996 until AD diagnosis |
|  | ICD-9: 2950-2959, 297 | 1987-1995 |
|  | ICD-8: 2950-2959, 297 | 1968-1986 |
| **Other psychosis** | Care register for health care |  |
|  | ICD-10: F302, F312, F315, F323, F333, F23, F28, F29 | From 1996 until AD diagnosis |
|  | ICD-9: 2962E, 2963E, 2964E, 2967, 2961E, 2988, 2989 | 1987-1995 |
|  | ICD-8: 2961-2969, 290, 2980, 2981, 2982, 2983, 2988, 2989, 299 | 1968-1986 |
| **Non-psychotic bipolar disorder** | Care register for health care |  |
|  | ICD-10: F300-F301, F303-F311, F313-F314, F316-F319 | From 1996 until AD diagnosis |
|  | ICD-9: 2962A-D, 2962F-G, 2963A-D, 2963F-G, 2964A-D, 2964F-G, 2968, 2969 | 1987-1995 |
|  | ICD-8: - |  |
| **Non-psychotic depression** | Care register for health care |  |
|  | ICD-10: F320-F322, F324-F332, F334-F339, F341, F3810 | From 1996 until AD diagnosis |
|  | ICD-9: 2961A-D, 3004 | 1987-1995 |
|  | ICD-8: 3004, 7902 | 1968-1986 |
| **Other mood disorder** | Care register for health care |  |
|  | ÏCD-10: F340, F342-F3809, F3811-F39 | From 1996 until AD diagnosis |
|  | ICD-9: - |  |
|  | ICD-8: - |  |
| **Any other psychiatric disorder (including those which are not mentioned above)** | Care register for health care |  |
|  | ICD-10: F10-F69, F90-F99 | From 1996 until AD diagnosis |
|  | ICD-9: 295-298, 300-301, 3071-3075, 312, 313-314 | 1987-1995 |
|  | ICD-8: 295-301, 303-308, 7902 | 1968-1986 |
| **Antidepressant use** | Prescription register |  |
|  | ATC code N06A | 1995 until AD diagnosis |
| **Antipsychotic use** | Prescription register |  |
|  | ATC code N05A (excluding N05AN and N05AB04) | 1995 until AD diagnosis |
| **Opioid use** | Prescription register |  |
|  | ATC code N02A | 1995 until AD diagnosis |
| **Benzodiazepine and related drugs use** | Prescription register |  |
|  | ATC codes N05BA, N05CD, N05CF, N03AE01 | 1995 until AD diagnosis |
| **Occupational social class** | Highest occupational social class, Statistics Finland classification (please see Kalamägi et al, Annals of Medicine 2019*) | 1972 until AD diagnosis |

COPD = Chronic obstructive pulmonary disease, AD = Alzheimer’s disease

*Kalamägi J, Lavikainen P, Taipale H, et al. Predictors of high hospital care and medication costs and cost trajectories in community‐dwellers with Alzheimer’s disease. Ann Med. 2019;51:294‐305. doi:10.1080/07853890.2019.1642507
